# Xylan-Modified-Based Hydrogels with Temperature/pH Dual Sensitivity and Controllable Drug Delivery Behavior

**DOI:** 10.3390/ma10030304

**Published:** 2017-03-16

**Authors:** Wei-Qing Kong, Cun-Dian Gao, Shu-Feng Hu, Jun-Li Ren, Li-Hong Zhao, Run-Cang Sun

**Affiliations:** 1 State Key Laboratory of Pulp and Paper Engineering, South China University of Technology, Guangzhou 510640, China; kongweiqing1119@gmail.com (W.-Q.K.); gaocundian@gmail.com (C.-D.G.); shufengbeyond@126.com (S.-F.H.); zhaolh@scut.edu.cn (L.-H.Z.); 2 Beijing Key Laboratory of Lignocellulosic Chemistry, Beijing Forestry University, Beijing 100083, China; rcsun3@bjfu.edu.cn

**Keywords:** **Keywords:** modified xylan, hydrogels, temperature/pH dual sensitivity, drug controlled release, biocompatibility

## Abstract

Among the natural macromolecules potentially used as the scaffold material in hydrogels, xylan has aroused great interest in many fields because of its biocompatibility, low toxicity, and biodegradability. In this work, new pH and thermoresponsive hydrogels were prepared by the cross-linking polymerization of maleic anhydride-modified xylan (MAHX) with *N*-isopropylacrylamide (NIPAm) and acrylic acid (AA) under UV irradiation to form MAHX-*g*-P(NIPAm-co-AA) hydrogels. The pore volume, the mechanical properties, and the release rate for drugs of hydrogels could be controlled by the degree of substitution of MAHX. These hydrogels were characterized by swelling ability, lower critical solution temperature (LCST), Fourier-transform infrared (FTIR), and SEM. Furthermore, the cumulative release rate was investigated for acetylsalicylic acid and theophylline, as well as the cytocompatibility MAHX-based hydrogels. Results showed that MAHX-based hydrogels exhibited excellent swelling–deswelling properties, uniform porous structure, and the temperature/pH dual sensitivity. In vitro, the cumulative release rate of acetylsalicylic acid for MAHX-based hydrogels was higher than that for theophylline, and in the gastrointestinal sustained drug release study, the acetylsalicylic acid release rate was extremely slow during the initial 3 h in the gastric fluid (24.26%), and then the cumulative release rate reached to 90.5% after sustained release for 5 h in simulated intestinal fluid. The cytotoxicity experiment demonstrated that MAHX-based hydrogels could promote cell proliferation and had satisfactory biocompatibility with NIH3T3 cells. These results indicated that MAHX-based hydrogels, as new drug carriers, had favorable behavior for intestinal-targeted drug delivery.

## 1. Introduction

Controlled drug-delivery systems were designed to deliver drugs at desirable times and/or to specific sites for achieving a therapeutic purpose [1]. For developing drug-delivery carriers, the major challenges were preparation of a suitable biomaterial that could ensure an excellent drug-releasing rate at the required dose to a target location, while also being nontoxic with good biocompatibility [2]. In recent years, considerable attention has been paid to the synthesis of intelligent hydrogels for the purpose of drug delivery because of their responsiveness to external stimuli such as pH, solvent composition, ionic strength, temperature and light, and other factors [3,4].

Temperature- and pH-sensitive hydrogels represent the most investigated class of intelligent hydrogels, and have been used extensively [5,6,7,8,9]. Variations in pH occur at several body sites, such as the gastrointestinal tract, blood vessels, and vagina, and therefore suitable pH- and temperature-responsive hydrogels are needed for drug release. Temperature- and pH-sensitive hydrogels can protect a drug from enzymatic hydrolysis or being destroyed by stomach acid, and therefore they can be considered as the ideal candidate for a drug-delivery system [10]. Nevertheless, it should be noted that most traditional temperature- and pH-sensitive hydrogels generally have several limitations. Firstly, the traditional hydrogels usually suffer from poor biodegradability and biocompatibility. Secondly, the drug-loading and encapsulation efficiency are limited. Finally, another serious limitation of the normal hydrogel as a potential drug carrier is its poor sustained release capability [11]. Therefore, for the preparation of hydrogels possessing nontoxicity, biocompatibility, high drug-loading and encapsulation efficiency, and controlled drug delivery, it is very necessary to change their structure.

In recent years, natural polymer-based hydrogels have aroused broad interests because of their unique adjustable structure, and properties such as the low cost and good biodegradability and biocompatibility [3,12,13,14,15]. Hemicellulose, a renewable plant polysaccharide, ranks second to cellulose in terms of lignocellulosic biomass content, and accounts for 1/4 to 1/3 of agriculture residues [16]. Due to the low cost and good biodegradability and biocompatibility, the high value-added utilization of hemicellulose has attracted much attention [17]. Xylan, as the most common hemicellulose, has unique physiological properties, such as inhibition of cell mutation; promotion of cell adhesion, proliferation, and innate immunological defense; and anticancer effects. These properties make xylan suitable for preparation of hydrogels used in drug release and biomedical engineering [8,18]. Moreover, hydroxyl and carboxylic groups on the xylan chains provide more opportunities for chemical or enzymatic modifications, while maintaining their native structure. Especially, the introduction of carboxyl groups and unsaturated double bonds can improve the pH response performance and the reaction efficiency of hemicellulose-based hydrogels, thereby extending their application in drug release. Maleic anhydride (MAH), as a small molecule of multifunctional groups, can react chemically with carbohydrate hydroxyls to form polymeric intermediates–MAH derivative, which contains both carboxyl groups and unsaturated double bonds. The carboxyl groups and double bonds in these derivatives can improve the pH response and adjust the cross-linking density of hydrogels and thus can be used as functional groups in drug delivery [19,20]. Therefore, MAH-modified xylan (MAHX) can also be imparted with potential applications in the preparation of hydrogels for drug delivery.

In this work, new pH- and thermoresponsive hydrogels were prepared by the cross-linking polymerization of MAHX with *N*-isopropylacrylamide (NIPAm) and acrylic acid (AA) using *N*, *N*’-methylene-bis-acrylamide (MBA) as the cross-linker under UV irradiation to form MAHX-*g*-P(NIPAm-co-AA) hydrogels, and their application in the drug-release system was studied. The aim of this study was to improve the encapsulation efficiency and regulate the drug controlled release behaviors of hydrogels by changing the degree of substitution (DS) of MAHX and the hydrogels’ structure. The physical and chemical properties of hydrogels were characterized by the swelling ability, the lower critical solution temperature (LCST), scanning electron microscopy (SEM) and Fourier-transform infrared spectroscopy (FTIR). Acetylsalicylic acid is widely used as a model compound because of its prevention of and therapy for thrombotic diseases, and it represents structures which form parts of many drug substances (benzene ring and carboxyl group) [21]. Theophylline, belonging to alkaloids, is a bronchodilator widely used in asthma therapy, and can exist either as the anhydrate or the monohydrate depending on the conditions under which it is stored [22]. The two compounds were applied as drug models for the study of drug-delivery performance of prepared hydrogels. Moreover, the cytocompatibility of MAHX-based hydrogels was studied by MTT methods.

## 2. Experimental Section

### 2.1. Materials

Beech wood xylan (*M*_w_ of 130,000 g·mol^−1^) was obtained from Sigma Aldrich, Germany. 1-Butyl-3-methylimidazoliumchloride ([BMIM]Cl) ionic liquids (ILS) were bought from Lanzhou Greenchem ILS, LICP. CAS (Lanzhou, China). MBA (98%) and acetylsalicylic acid (99%) were supplied by Aladdin Reagent Company Limited (Shanghai, China). LiOH, maleic anhydride (A. P.), and theophylline were purchased from Shanghai Macklin Biochemical Co., Ltd. (Shanghai, China). NIPAm (98%), AA (98%), 2,2-dimethoxy-2-phenylacetophenone (DMPA, 99%) *N*-methyl pyrrolidone (NMP, 99%) were obtained from Guangzhou Chemical Reagent Factory. The NIH3T3 cells were supplied by School of Chemical Engineering, Jinan University (Guangzhou, China), 3-(4,5-dimethylthiazol-2-yl)-2,5-diphenyltetrazoliumbromide (MTT) was supplied by Sigma-Aldrich (St. Louis, MO, USA), and fetal bovine serum (FBS) was supplied by SiJiqing Bio-engineering Material Company (Hangzhou, China). All chemical reagents were analytical reagent grade.

### 2.2. Preparation of MAHX or MAHX-Based Hydrogels

#### 2.2.1. Preparation of MAHX

MAHX was synthesized according to our previous work [23]. Dry xylan (0.33 g) was dissolved into [BMIM]Cl ionic liquids (2.5%, *w*/*w*) to form the mixed solution. The mixture was stirred at 90 °C for 4 h under the protection of nitrogen to guarantee complete dissolution of xylan. Then, 0.0025 g of LiOH and the required amount of MAH (Table 1) were added and stirred for 80 min at 80 °C. After the required time, the mixture was cooled to room temperature, precipitated with 95% (*w*/*w*) ethanol, and centrifuged at 4000 rpm for 30 min. The precipitate was washed by 95% (*w*/*w*) ethanol. In the end, obtained MAHX was dried at 45 °C in a vacuum oven for 16 h. The DS of MAHX was determined according to the method in our previous work [23].

#### 2.2.2. Preparation of MAHX-Based Hydrogels

MAHX-based hydrogels were synthesized by the cross-linking polymerization under UV irradiation. The detailed synthesis conditions are summarized in Table 1. Briefly, MAHX with different DS (in Table 1) was dissolved in distilled water at a concentration of 5% (*w*/*w*) and stirred until MAHX was completely dissolved. Under nitrogen atmosphere, a certain amount of NIPAm, AA, and MBA were added, respectively. Photoinitiator (DMPA 5% *w*/*w*, based on MAHX weight) dissolved in the NMP solution (2.5% *w*/*w*) was added to the mixed solution. Then, the solution was transferred into a glass beaker with a diameter of 5 cm and irradiated under ultraviolet light (365 nm and 40 W) at the intensity of ~199.9 mW/cm^2^ [24,25] for 6 h at room temperature. The obtained hydrogels were washed thoroughly in 250 mL deionized water for 5 days to remove unreacted chemicals, and the used deionized water was changed twice a day. Finally, the obtained hydrogels were freeze-dried (105 Torr, 48 h, and −70 °C).

### 2.3. The Swelling Behavior Studies of Hydrogels

Swelling studies of the MAHX-based hydrogels were carried out by the gravimetric method. The dried hydrogels were swollen in buffer solutions with different pH values (pH 1.5 and pH 7.4) at different temperatures (from 25 °C to 37 °C), respectively, to reach the equilibrium swelling state. The ionic strength of the buffer solution was adjusted by NaCl to keep it constant at 0.5 M. All samples were measured three times and the average taken. The design formula of the equilibrium swelling ratios (*S_eq_*) is shown as follows [26]:

*S_eq_* = (*W_eq_* − *W_d_*)/*W_d_* (1)

where *W_eq_* and *W_d_* are the weight of swelling hydrogels and dried hydrogels, respectively.

### 2.4. Compression Test of Hydrogels

Compressive stress measurement was taken using an Instron Universal Testing Machine 5565. Hydrogels were cut into columns with a diameter of 20 mm and height of 20 mm using scalpels. The testing was carried out with a speed of 2 mm/min until the compression ratio reached to 50% in a room with constant temperature and humidity (25 °C and 50% humidity).

### 2.5. Characterization

SEM (Hitachi S3700, Tokyo, Japan) with an acceleration voltage of 10 kV was carried out to study and compare the surface morphology of xylan, MAHX, and MAHX-based hydrogels. FTIR spectra of samples were recorded in KBr pellets on Nicolet 750 (Sarasota, FL, USA) and the wavelength ranged from 4000 to 400 cm^−1^. Differential scanning calorimetry (DSCQ200, TA Instrument, Wilminton, NJ, USA) analysis was utilized for studying the LCST of hydrogels. The thermal analyses were performed from 15 °C to 55 °C on the swollen hydrogels under a dry nitrogen atmosphere with a flow rate of 25.0 mL/min and a heating rate 5 °C/min.

### 2.6. Loading of Acetylsalicylic Acid and Theophylline into Hydrogels

The dried hydrogels were submerged in ethanol solution containing 10% (*w*/*v*) acetylsalicylic acid and theophylline, respectively, and stirred for 24 h; loading was performed under the dark environment [27]. Then, hydrogels were dried in a vacuum oven. The weight was recorded when the drug-loaded hydrogels reached a constant weight. The content of acetylsalicylic acid and theophylline in hydrogels and the encapsulation efficiency were estimated by the following equations [28]:

Drug loading (%) = *W_d_*/*W_h_* × 100 (2)

Encapsulation efficiency (%) = *W_a_*/*W_m_* × 100 (3)

where *W_d_* and *W_h_* represent the total weight of drug in hydrogels and the total weight of dried hydrogels, respectively. *W_a_* and *W_m_* refer to the actual drug-loading weight and the theoretical maximum drug-loading weight, respectively.

### 2.7. In Vitro Drug Release

The dried hydrogels were ground into powder and then measurements were carried out with a physical property measurement system (PPMS-9, Quantum Design Inc., San Diego, CA, USA) at room temperature. The release behavior of drug-loaded hydrogels was characterized using a horizontal oscillator with a shaking speed of 50 rpm at 37 °C. The drug-loaded hydrogels were immersed into the artificial gastric liquid (pH = 1.2, HCl–NaCl buffer solution) and the artificial intestinal liquid (pH = 7.4, Tris–NaCl buffer solution), respectively. Five milliliters of each buffer solution was collected at 1 h intervals. The amount of acetylsalicylic acid and theophylline was determined by a UV/V spectrophotometer (SHIMADZU UV1800, Kyoto, Japan) at 296 nm and 270 nm, respectively. Meanwhile, a volume of medium equal to the solution removed for testing was added back to maintain a constant volume. All samples were measured three times for in vitro release tests and the average taken. The percentage of drug released was estimated as follows:

Cumulative release (%) = *W_dt_/W_∞_* × 100% (4)

where *W_dt_* is the weight of drug at time t and *W_∞_* is the total weight of loaded drug in hydrogels, respectively.

### 2.8. Cell Viability Study

The cell viability assay was conducted by MTT method using NIH3T3 cells to measure the cell proliferation and the cell activity in the solution including prepared hydrogel powder (gel-3). NIH3T3 cells (2 × 10^3^ cells per well) were seeded in 96-well plates (100 µL) and cultivated for 24 h. Then, different concentrations of hydrogel samples (1.6, 0.8, 0.4, 0.2, 0.1, and 0.05 mg/mL) were put into the cells and cultivated for 24 h and 72 h. Then, the medium was removed and 30 μL MTT (5 mg/mL) was added, followed by 4 h of incubation at 37 °C. Then, the supernatant was removed, and 150 µL of DMSO was added to dissolve the formazan precipitate. The absorbance of the solution was measured by microplate reader (Bio-Rad 550, Hercules, CA, USA) at 570 nm to determine the optical density (OD) value. Each group was measured three times and the average taken. The cell viability was calculated by following formula [29]:

Cell viability = *OD_gel_*/*OD_ctrl_* × 100% (5)

where *OD_gel_* is the optical density of cells cultured on hydrogels and *OD_ctrl_* is the optical density of the control group.

## 3. Results and Discussion

### 3.1. Temperature and pH-Dependent Swelling Behaviors of Hydrogels 

**Figure 1:**
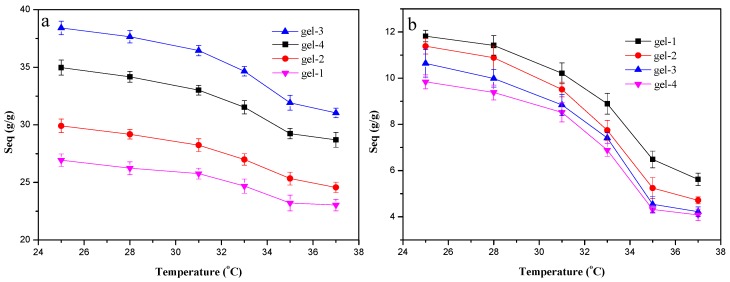
The influences of the temperature (25–37 °C) and pH (7.4 (**a**) and 1.5 (**b**)) on the swelling ratio (S_eq_) of MAHX-gels.

The swelling ability determined the physical properties and applications of hydrogels. To study the influence of the hydrogel composition (DS of MAHX) and environment conditions (temperature and pH) on the swelling ratio of hydrogels, a systematic study on the swelling characteristic was carried out and the results are shown in Figure 1. The S_eq_ values of MAHX-based hydrogels (MAHX-gels) in pH 7.4 at different temperatures were illustrated in Figure 1a. Obviously, the S_eq_ was affected by the temperature and the DS of MAHX. The S_eq_ of all the samples were decreased with the increase of temperature from 25 °C to 37 °C. Besides, the fastest decline in the S_eq_ of hydrogels occurred from 33 °C to 35 °C, revealing that the LSCT of hydrogels was distributed between 33 °C and 35 °C. This could be explained by the fact that when the temperature was above the LCST, NIPAm moieties became dehydrated, leading to the shrinkage of the network of hydrogels and thus decreasing the swelling ratio [30]. The S_eq_ of hydrogels at 37 °C in pH 7.4 firstly increased from 23.03 g/g to 31.03 g/g with the increase of the DS of MAHX from 0.12 (gel-1) to 0.48 (gel-3). However, further increasing the DS of MAHX from 0.48 (gel-3) to 0.65 (gel-4) caused the swelling ratio to decrease. The hydrophilicity of hydrogels could be improved by the modification of xylan and be enhanced by increasing the DS of MAHX and, consequently, increasing the swelling ratio. Meanwhile, when xylan was modified by MAH, due to the introduction of carboxyl groups, many highly active carbon–carbon double bonds were introduced into the xylan backbone. During the hydrogel formation, these carbon–carbon double bonds could cross-link with AA and NIPAm, resulting in a higher cross-link density, reducing the pore diameter of hydrogels, and thus decreasing the swelling ratio [31].

As shown in Figure 1b, the S_eq_ of MAHX-gels decreased faster in pH 1.5 than in pH 7.4 (Figure 1a). This was because the existence of many hydroxyl and carboxyl groups in the hydrogel network promoted the formation of hydrogen bonds, thereby inhibiting the expansion of the network of hydrogels in the acidic system. The difference was that the S_eq_ of MAHX-gels decreased with the increase of the DS of MAHX. This could be explained that the content of the carboxyl groups was increased with the increase of the DS of MAHX, resulting in stronger hydrogen-bonding interaction and higher cross-link density, thus decreasing the swelling ratio.

### 3.2. The Swelling and Deswelling Behaviors of Hydrogels in Buffers 

**Figure 2:**
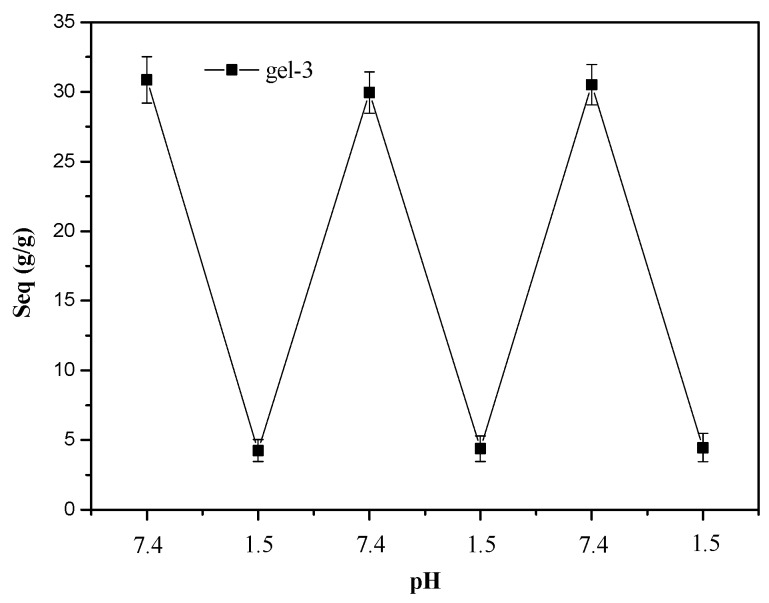
Reversible swelling–deswelling behavior of gel-3 in the buffer solutions with pH 1.5 and 7.4 at 37 °C.

The swelling and deswelling behavior of hydrogels are important parameters used to evaluate its reversibility and durability. Figure 2 illustrates reversible swelling–deswelling behaviors of gel-3 in the buffer solutions with pH 1.5 and 7.4 at 37 °C. When the swollen gel-3 in the buffer solutions (pH 7.4) was transferred into the solution of pH 1.5, the S_eq_ of gel-3 was sharply decreased from 30.86 g/g to 4.25 g/g, 29.95 g/g to 4.38 g/g, and 30.52 g/g to 4.45 g/g, respectively. At a low pH region, the carboxylates (–COO–) in gel-3 were generally protonated to become COOH and thus led to the deswelling of the hydrogel. However, when the hydrogel was transferred into the solution of pH 7.4 again, the COOH became ionized, and the resulting electrostatic repulsion in the network led to the reswelling of hydrogels [32]. After three swelling–deswelling cycles, there was no obvious decrease in the swelling ratio of gel-3 in either pH 1.5 or pH 7.4. This indicated that hydrogels had good durability and reversibility, implying that these hydrogels have potential application in drug-delivery systems. A similar result was also reported for other hemicellulose-based poly (acrylic acid) hydrogels [33,34].

The reversible swelling–deswelling behaviors of gel-1, gel-2, and gel-4 are displayed in Figure S1 (Supporting Information file) in buffer solutions at pH 1.5 and 7.4 at 37 °C. After three swelling and deswelling cycles, there was no obvious decrease in the swelling ratio of gel samples in either pH 1.5 or pH 7.4, which indicated that hydrogels had good durability and reversibility. 

### 3.3. Morphological Analysis

**Figure 3:**
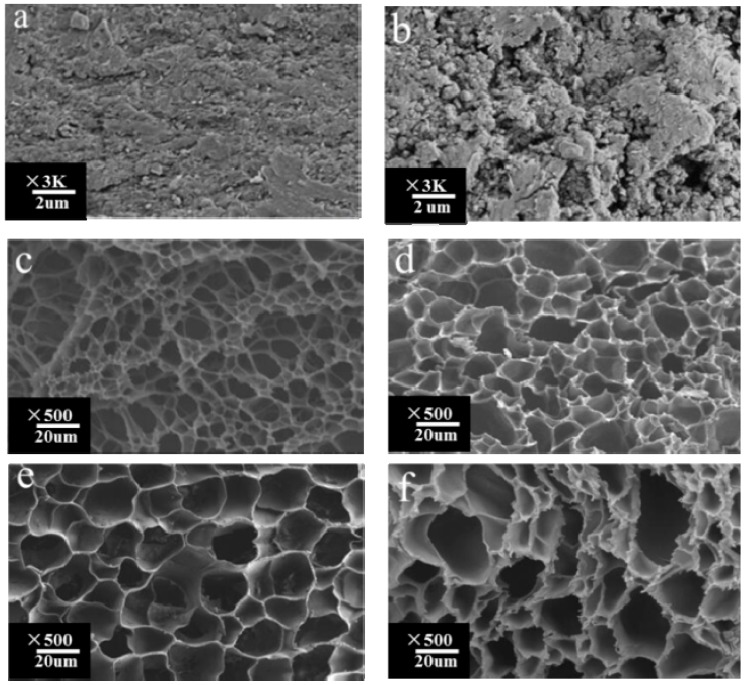
SEM images of xylan (**a**); MAHX (**b**); and freeze-dried gel-1 (**c**); gel-2 (**d**); gel-3 (**e**); and gel-4 (**f**). The hydrogel samples swelled at 37 °C and freeze-dried before determination.

Figure 3 displays the SEM images of xylan (a); MAHX (b); and freeze-dried gel-1 (c); gel-2 (d); gel-3 (e); and gel-4 (f). Compared with the native xylan (Figure 3a), the surface of MAHX (Figure 3b) became coarser due to the attachment of MAH to the xylan backbone, which led to the change in the structural morphology of xylan. All the hydrogel samples displayed a honeycomb-like architecture. Moreover, the pore volume of hydrogels was increased with the increase of the DS of MAHX. The hydrophilicity of hydrogels was increased with the increase of the DS of MAHX, resulting in a larger swelling ratio, and thus increasing the pore volume. In addition, compared to gel-1 and gel-2, the network structure of gel-3 was more uniform. This was because the cross-link density of the hydrogel was increased with the increase of the DS of MAHX, resulting in a cross-link density perfect for forming the cross-linking network. However, further increasing the DS of MAHX caused the continued increase in the cross-link density of hydrogels, thereby resulting in an uneven crosslinking density, which induced a nonuniform network of gel-4. Interestingly, compared to the other gels (gel-1, gel-2, and gel-4), gel-3—with even larger pores and thinner walls—also had the highest drug-loading and encapsulation efficiency, as will be discussed. The same conclusions were obtained by Cao et al. and Zhao et al. [33,35]. Thus, the gel-3 was the desirable candidate for drug delivery.

Table 2 shows the mechanical properties of the observed hydrogels. The compress stress firstly increased from 53.34 kPa to 68.25 kPa with the increase of the DS of MAHX from 0.12 (gel-1) to 0.48 (gel-2). Further increasing the DS of MAHX from 0.48 (gel-1) to 0.65 (gel-4) resulted in the compress stress decreasing from 68.25 kPa to 67.56 kPa. The change trend of elasticity modulus for hydrogels was the same with the compress stress, and gel-3 had the highest compression strength and elasticity modulus, as shown in Table 2. Obviously, a denser network was observed with the increase of the DS of MAHX, thus, resulting in stronger compression strength. However, further increasing the DS of MAHX caused the continued increase in the cross-link density of hydrogels, consequently resulting in an uneven cross-linking density, which induced a vulnerable network of hydrogels [26]. This well agreed with the results obtained from the swelling ratio and SEM.

### 3.4. FTIR Spectra of Xylan, MAHX and Prepared Hydrogels

**Figure 4:**
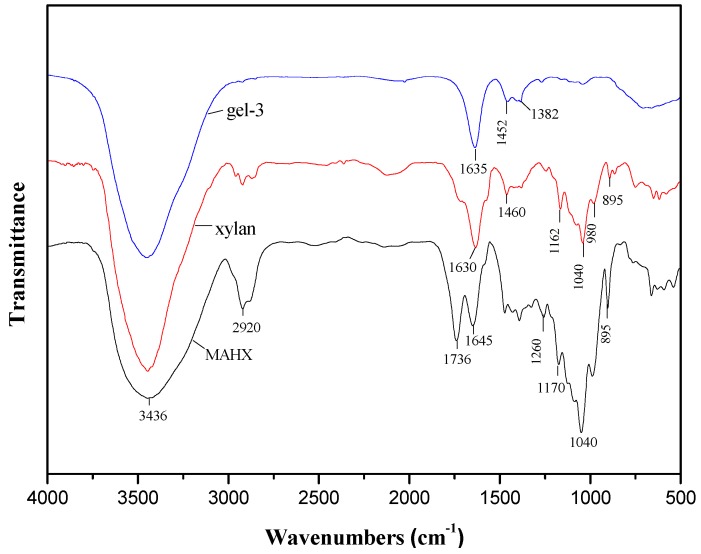
Fourier-transform infrared (FTIR) spectra of xylan, MAHX, and gel-3.

Figure 4 illustrates the FTIR spectra of xylan, MAHX, and gel-3. In the spectrum of xylan, the absorption at 3436 cm^−1^, 2920 cm^−1^, 1630 cm^−1^, 1460 cm^−1^, 1162 cm^−1^, 1040 cm^−1^, 980 cm^−1^, and 895 cm^−1^ are characteristic absorption bands of xylan [34,36]. The bands at 2923 cm^−1^ and 1040 cm^−1^ are assigned to the C–H and C–O–C stretching vibration band of xylan, respectively. A sharp band at 895 cm^−1^ is associated with β-glycosidic linkages between the sugar units, indicating that the xylose residues forming the backbone of the macromolecule are linked by β-form bonds [37]. The wide absorbance at 3436 cm^−1^ is assigned to the hydroxyl stretching vibration of xylan. Compared with xylan, the absorption at 3436 cm^−1^ of MAHX and gel-3 decreased significantly, which indicated that esterification had occurred between the hydroxyl groups in xylan and the anhydride groups of the MAH. In the spectrum of MAHX, there are a few new bands compared with the spectrum of xylan in Figure 4. The bands at 1736 and 1170 cm^−1^ are assigned to carbonyl stretching vibrations of C=O in anhydride groups and the C–O vibration in MAH, respectively [19], indicating the successful attachment of MAH onto xylan [23,26]. In the spectrum of gel-3, the bonds at 1635 cm^−1^ and 1560 cm^−1^ are assigned to stretching vibration of –N–H in NIPAm [24,38,39,40]. The bonds at 1452 cm^−1^ and 1382 cm^−1^ are assigned to the asymmetric absorption peak of COO^−^ and bending vibration of C–H originating from AA, respectively [41]. These signal changes observed in the FTIR spectra confirmed the successful synthesis of the target hydrogels.

### 3.5. LCST of Hydrogels

**Figure 5:**
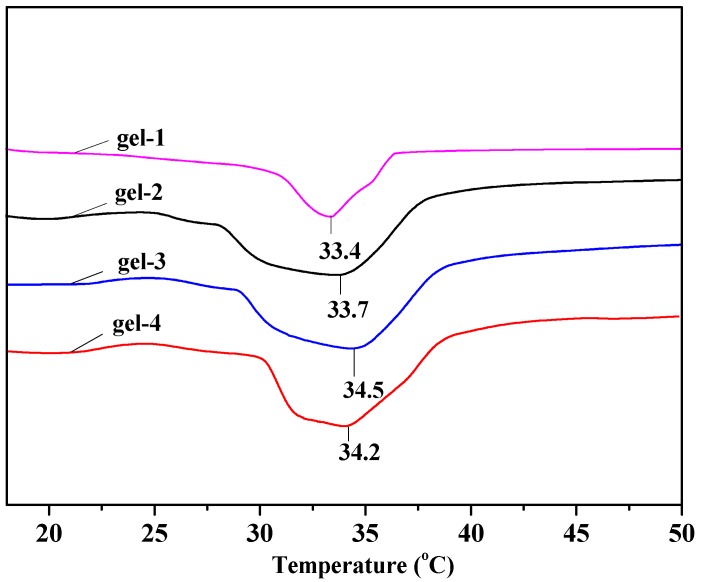
Differential scanning calorimetry (DSC) curves of hydrogels.

Figure 5 exhibits DSC heating scans of gel-1, gel-2, gel-3, and gel-4. The LCST of gel-1, gel-2, gel-3, and gel-4 emerged at 33.4 °C,33.7 °C,34.5 °C, and 34.2 °C, respectively. The LCST of hydrogels firstly increased with the increase of the DS of MAHX and then decreased. Gel-3 showed the highest LCST of 34.5 °C nearest to body temperature. It could be deduced that the hydrophilicity of the hydrogel networks increased with the increase of the DS of MAHX, increasing the combination of intermolecular forces, especially the hydrogen-bonding interaction between the hydrogel matrixes and water molecules in the system. This implied that breaking of hydrogen bonds required more energy, which made the transition temperature of LCST become higher and broader [42]. Nevertheless, further increasing the DS of MAHX led to the nonuniform cross-linking of the hydrogel network, which led to the decrease in the LCST. Therefore, the DS of MAHX used to prepare hydrogels had an important effect on the LCST of hydrogels. Thus, gel-3 with LCST of 34.5 °C nearest to the body temperature was the most suitable as the carrier for drug controlled release in the biomedical field.

### 3.6. In Vitro Drug Release of Hydrogels

The drug-loading and encapsulation efficiency of gel-1, gel-2, gel-3, and gel-4 for acetylsalicylic acid are shown in Table 2. Both the drug-loading and encapsulation efficiency of hydrogels were firstly increased and then decreased with the increase of the DS of MAHX. Gel-3 had the highest drug-loading and encapsulation efficiency. The interpretation is that gel-3 had a more suitable pore diameter and ordered networks than gel-1 and gel-2, which provided more electrostatic interaction for acetylsalicylic acid, while the irregular internal structure of gel-4 had a lower affinity and electrostatic interaction than gel-3 [28]. Therefore, gel-3 was the most suitable candidate for the application as the carrier for drug controlled release.

**Figure 6:**
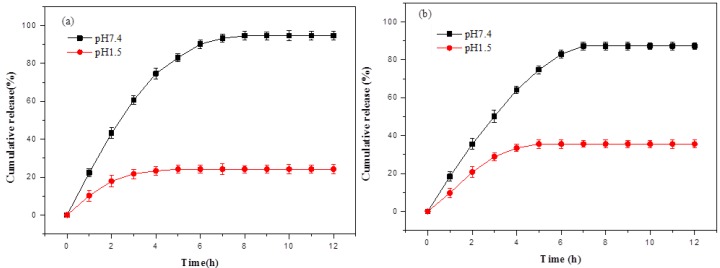
In vitro cumulative drug release from the drug-loaded gel-3 in simulated gastric and intestinal fluids at 37 °C: (**a**) acetylsalicylic acid and (**b**) theophylline.

To investigate whether gel-3 had special drug release behavior and whether the type of drug had an effect on the drug-release behavior of gel-3, acetylsalicylic acid and theophylline were used as model drugs for studying the in vitro release behavior of gel-3 at 37 °C. The cumulative acetylsalicylic acid and theophylline release at different pH conditions (pH 7.4 and pH 1.5) are shown in Figure 6. It was found that the release rate of acetylsalicylic acid from gel-3 in simulated intestinal fluids (pH 7.4) increased quickly during the initial 4 h and then slowed down and approached the release equilibrium (Figure 6a). The release equilibrium was achieved at 8 h and the maximum release percentage was 94.56%. While in the simulated gastric fluids (pH 1.2), gel-3 showed a slower and steadier acetylsalicylic acid release rate. The maximum release percentage (24.25%) was achieved within 4 h. The cumulative release value of acetylsalicylic acid in the simulated intestinal fluids (pH 7.4) was higher than that in the simulated gastric fluids (pH 1.5) during the releasing process. This could be attributed to the difference in the swelling ratios of hydrogels in different mediums.

Gel-3 showed the same trend for the release of theophylline (Figure 6b). The release equilibriums were achieved at 7 h in pH 7.4 (87.25%) and 5 h in pH 1.5 (35.6%), respectively. In comparison, gel-3 presented the better release behavior for the acetylsalicylic acid release than that for theophylline release. This could be attributed to the existence of –COOH in acetylsalicylic acid, which could adjust the release rate fast and flexibly in different pH solutions. When the gel was put into the solution of pH 7.4, these –COOH groups in acetylsalicylic acid would be ionized into –COO–, the electrostatic repulsion forces would induce the expansion of the hydrogel network, and, finally, result in a faster release rate. When the hydrogel was put into a lower pH value (pH=1.5), the –COO– groups would be generally protonated into –COOH with the pH decrease and further result in a slower release rate [33]. Besides, it might be speculated that the drug release behavior was controlled by both the performance of hydrogels and the nature of drugs.

**Figure 7:**
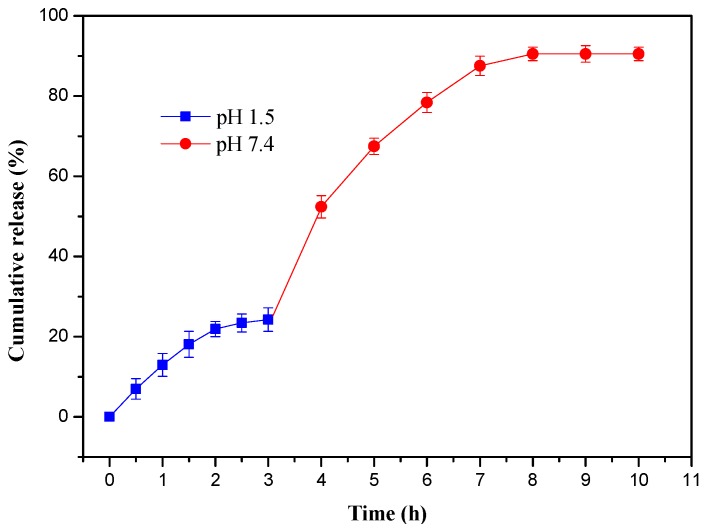
Acetylsalicylic acid release performance of gel-3 in pH 1.5 buffer solution for 3 h followed by the drug’s release in pH 7.4 for 10 h at 37 °C.

To study the sequential release behaviors, the acetylsalicylic acid-loaded hydrogel was firstly put into simulated gastric fluids (pH 1.5) for 3 h and then transferred into simulated intestinal fluids (pH 7.4) for 7 h, and the results are shown in Figure 7. The initial release of acetylsalicylic acid in simulated gastric fluids (pH 1.5) was fast during the first 2 h and then slowed down and achieved the release equilibrium within 3 h. The maximum release percentage was 24.26% at 3 h. In the simulated intestinal fluid (pH 7.4), the release rate increased rapidly and reached to 52.36% at 4 h, nearly double that of the first 3 h in pH 1.5. The release equilibrium was achieved at 8 h and the cumulative release percentage was 90.5%. Moreover, the chromogenic reaction between acetylsalicylic acid and ferric chloride (FeCl_3_) was carried out to test whether acetylsalicylic acid was hydrolyzed or not, which is displayed in Figure S2 (Supporting Information file). The results showed that no chromogenic reaction happened after dropping FeCl_3_ into the acetylsalicylic acid solution released by gel-3. This implied that acetylsalicylic acid was not hydrolyzed during the releasing process. Therefore, these hydrogels could efficiently control the release of drug in gastric fluids and intestinal fluids, and had the desired protective effect in the stomach for oral drug delivery. When hydrogels were transferred to the simulated intestinal fluid (pH 7.4), most of acetylsalicylic acids were released from the hydrogel network. Thus, prepared hydrogels had the intestinal-targeted drug-delivery function.

### 3.7. Cytocompatibility of Hydrogels

**Figure 8:**
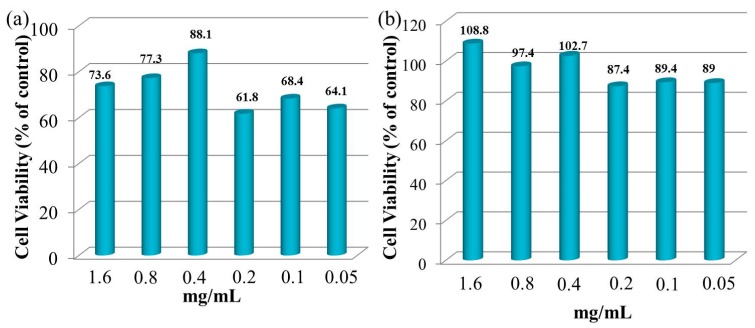
NIH3T3 cell viability of gel-3 under the different concentrations of 1.6–0.05 mg/mL after cultivating for 24 h and 72 h.

NIH3T3 cells were employed as model cells for evaluating the cytocompatibility of gel-3 by the MTT method [43]. Figure 8 illustrates the NIH3T3 cell viability under the different concentrations of gel-3 (0.05–1.6 mg/mL) after cultivating for 24 h and 72 h. After cultivating for 24 h, the cell viability of NIH3T3 cells was firstly increased from 73.6% to 88.1% with the decrease of the gel-3 concentration from 1.6 mg/mL to 0.4 mg/mL, and then decreased from 88.1% to 64.1% with further decreasing of gel-3 concentration from 0.4 mg/mL to 0.05 mg/mL. This could be interpreted as a higher concentration of hydrogel samples having an inhibitory effect on NIH3T3 cells at the beginning of the cultivating (24 h), while the hydrogel showed a significant decrease in cell survival at a lower concentration of hydrogel. This might be because the decrease of xylan content resulted in the decrease of cell compatibility. As expected, the cell viability of NIH3T3 cells was enhanced to some degree at different concentrations of gel-3 after cultivating for 72 h. These results confirmed that the xylan-based hydrogel had favorable biocompatibility with NIH3T3 cells. The same conclusions were obtained by Voepel et al. and Zhao et al. [35,44].

**Figure 9:**
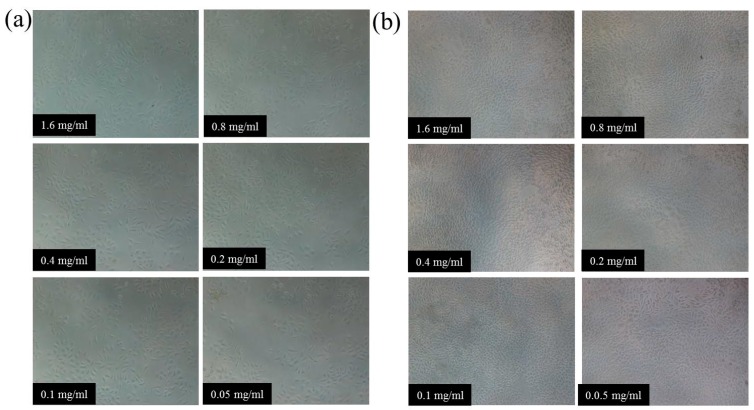
The micrographs of NIH3T3 cell distribution under the different concentrations of gel-3 samples: (**a**) cultivation for 24 h; (**b**) cultivation for 72 h.

Figure 9 illustrates the micrographs of NIH3T3 cell distribution under the different concentrations of gel-3 samples after cultivating for 24 h and 72 h. The NIH3T3 cells represented an aggregate distribution and they were relatively lower in number after cultivating for 24 h, while the NIH3T3 cells appeared to have a significant proliferation when they were distributed densely and uniformly after cultivating 72 h. The results were consistent with the cell viability shown in Figure 8. The observation and results above were indicative of the promising potential of xylan-based hydrogels in human drug-delivery fields because of their excellent biocompatibility.

## 4. Conclusions

In summary, new maleic anhydride modified xylan (MAHX)-based hydrogels with temperature/pH dual sensitivity and controllable drug delivery behavior were obtained by the cross-linking polymerization under UV light. These hydrogels presented good swelling–deswelling properties, honeycomb-like architecture, and temperature/pH dual sensitivity. Moreover, the pore volume, the mechanical properties, and the drug release rate of hydrogels could be controlled by the DS of MAHX. In vitro, the cumulative release rate of acetylsalicylic acid for MAHX-based hydrogels was higher than that of theophylline. Besides, in the gastrointestinal sustained drug release, the acetylsalicylic acid release rate was extremely slow at initial 3 h in the gastric fluid (24.26%), and then the cumulative release rate reached 90.5% after sustained release for 5 h in the stimulated intestinal fluid. Importantly, MAHX-based hydrogels had satisfactory biocompatibility with NIH3T3 cells. Therefore, MAHX-based hydrogels as drug carriers have potential application in human drug-delivery fields.

## Figures and Tables

**Table 1:** Degree of substitution (DS) of maleic anhydride-modified xylan (MAHX) and the preparation conditions of MAHX-based hydrogels.

Samples	MAH/xylan	DS of MAHX	NIPAm/MAHX (g/g)	AA/MAHX (g/g)	MBA/MAHX (g/g)
gel-1	1:1	0.12	0.1	10:1	0.1
gel-2	2:1	0.28	0.1	10:1	0.1
gel-3	4:1	0.48	0.1	10:1	0.1
gel-4	8:1	0.65	0.1	10:1	0.1

AA: acrylic acid; MBA: *N*,*N*’-methylene-bis-acrylamide; NIPAm: *N*-isopropylacrylamide.

**Table 2:** The compressive properties and drug-loading and encapsulation efficiency of hydrogels.

Samples	Compression Stress (kPa)	Elasticity Modulus (kPa)	Drug ^a^ Loading (%)	Encapsulation Efficiency (%)	LCST (°C)
gel-1	56.34 ± 3.68	78.54 ± 9.48	55.26	86.56	33.4
gel-2	60.42 ± 4.23	128.25 ± 6.56	58.2	89.62	33.7
gel-3	68.25 ± 3.34	168.67 ± 8.54	60.68	92.25	34.3
gel-4	67.56 ± 2.68	164.34 ± 8.78	58.45	90.56	34.2

LCST: lower critical solution temperature; ^a^ acetylsalicylic acid drug loading.

## Supplementary Materials

Supplementary Materials: The following are available online at www.mdpi.com/1996-1944/10/3/304/s1. Figure S1. Reversible swelling-deswelling behaviors of gel-1, gel-2 and gel-4 in the buffer solutions of pH 1.5 and 7.4 at 37 °C. Figure S2. The color change in the acetylsalicylic acid solution released by gel-3 before (a) and after (b) the addition of FeCl.
