# Supplementary material for: Xylan-Modified-Based Hydrogels with Temperature/pH Dual Sensitivity and Controllable Drug Delivery Behavior
Source: Materials (Basel). 2017 Mar 16;10(3):304. doi: 10.3390/ma10030304 (PMC5503382; doi:10.3390/ma10030304)
Supplement: Supplementary file 1 [file materials-10-00304-s001.pdf]

Supplementary Materials: Xylan-Modified based Hydrogels with Temperature/pH Dual Sensitivity and Controllable Drug Delivery Behavior

Wei-Qing Kong, Cun-Dian Gao, Shu-Feng Hu, Jun-Li Ren, Li-Hong Zhao and Run-Cang Sun

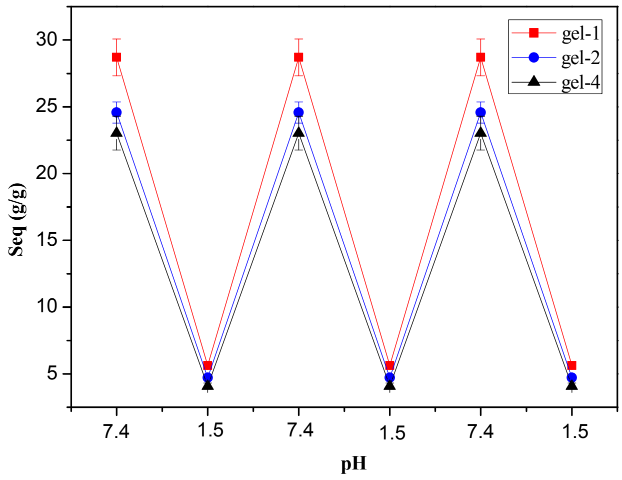

Figure S1. Reversible swelling-deswelling behaviors of gel-1, gel-2 and gel-4 in the buffer solutions of pH 1.5 and 7.4 at 37 °C.

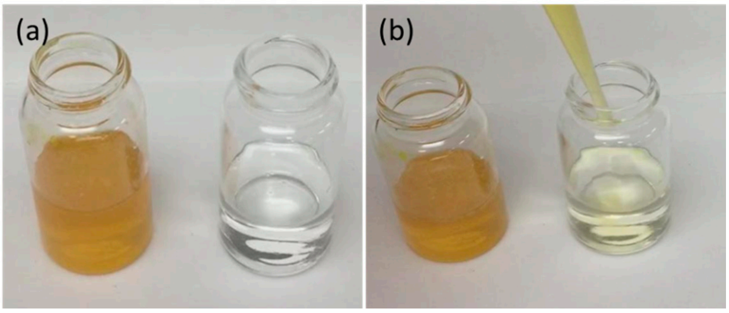

Figure S2. The color change in the acetylsalicylic acid solution released by gel-3 before (**a**) and after (**b**) the addition of $FeCl_3$.

© 2017 by the authors. Submitted for possible open access publication under the terms and conditions of the Creative Commons Attribution (CC BY) license (http://creativecommons.org/licenses/by/4.0/).
